# A targeted likelihood estimation comparing cefepime and piperacillin/tazobactam in critically ill patients with community-acquired pneumonia (CAP)

**DOI:** 10.1038/s41598-024-64444-3

**Published:** 2024-06-11

**Authors:** Cristian C. Serrano-Mayorga, Sara Duque, Elsa D. Ibáñez-Prada, Esteban Garcia-Gallo, María P. Rojas Arrieta, Alirio Bastidas, Alejandro Rodríguez, Ignacio Martin-Loeches, Luis F. Reyes

**Affiliations:** 1 Unisabana Center for Translational Science, Universidad de La Sabana, Chía, Colombia; 2 School of Medicine, Universidad de La Sabana, Chía, Colombia; 3 Clinica Universidad de La Sabana, Chía, Colombia; 4 Engineering Faculty, Universidad de La Sabana, Chía, Colombia; 5 ICU Hospital , Universitario de Tarragona Joan XXIII – IISPV – Universidad Rovira and Virgili - CIBERES, Tarragona, Spain; 6 Department of Intensive Care Medicine, Multidisciplinary Intensive Care Research Organisation (MICRO), St James’s Hospital, Dublin, Ireland; 7 Trinity College Dublin, Dublin, Ireland; 8 CIBER of Respiratory Diseases (CIBERES), Institute of Health Carlos III, Madrid, Spain; 9 Pulmonary Department, Hospital Clinic, Universitat de Barcelona, IDIBAPS, ICREA, Barcelona, Spain; 10 Pandemic Sciences Institute, University of Oxford, Oxford, UK

**Keywords:** Community-acquired pneumonia (CAP), Intensive care unit (ICU), Cefepime, Piperacillin/tazobactam, Targeted maximum likelihood estimation (TMLE), Infectious diseases, Antibiotics

## Abstract

Cefepime and piperacillin/tazobactam are antimicrobials recommended by IDSA/ATS guidelines for the empirical management of patients admitted to the intensive care unit (ICU) with community-acquired pneumonia (CAP). Concerns have been raised about which should be used in clinical practice. This study aims to compare the effect of cefepime and piperacillin/tazobactam in critically ill CAP patients through a targeted maximum likelihood estimation (TMLE). A total of 2026 ICU-admitted patients with CAP were included. Among them, (47%) presented respiratory failure, and (27%) developed septic shock. A total of (68%) received cefepime and (32%) piperacillin/tazobactam-based treatment. After running the TMLE, we found that cefepime and piperacillin/tazobactam-based treatments have comparable 28-day, hospital, and ICU mortality. Additionally, age, PTT, serum potassium and temperature were associated with preferring cefepime over piperacillin/tazobactam (OR 1.14 95% CI [1.01–1.27], *p* = 0.03), (OR 1.14 95% CI [1.03–1.26], *p* = 0.009), (OR 1.1 95% CI [1.01–1.22], *p* = 0.039) and (OR 1.13 95% CI [1.03–1.24], *p* = 0.014)]. Our study found a similar mortality rate among ICU-admitted CAP patients treated with cefepime and piperacillin/tazobactam. Clinicians may consider factors such as availability and safety profiles when making treatment decisions.

## Introduction

Community-acquired pneumonia (CAP) is one of the leading causes of death due to infectious diseases worldwide^1^. Almost 100,000 patients per year are admitted to intensive care units (ICU) due to CAP^2^. Overall, CAP is the fourth cause of death globally and the second cause of death in low-middle-income countries (LMIC)^1,3^. The mortality rate in CAP patients admitted to the ICU has remained steady during the last years and varies from 20 to 50%^4^. The economic burden attributable to the treatment of CAP exceeds €10 billion in Europe every year and more than $17 billion in the USA^5^. Thus, CAP is a growing healthcare problem that needs immediate attention, especially in patients admitted to the ICU.

Antibiotic treatment is the cornerstone for CAP patients^6,7^. It has been suggested that the reduction of 30-day mortality is strongly linked to broad-spectrum antibiotics and early-goal-directed treatment^8^. However, there is controversy about the best empirical antimicrobial treatment in patients admitted to the ICU due to CAP^9–11^. Current international guidelines recommend choosing the empiric antibiotic treatment for CAP patients based on individual risk factors for multidrug-resistant pathogens (MDRP) and local epidemiology. The empirical treatment recommended for patients with CAP who require admission to the ICU is a beta-lactam-based treatment along with a macrolide or quinolone^8^. However, this treatment is inadequate for more difficult-to-treat pathogens, such as *Pseudomonas aeruginosa* and Methicillin-Resistant *Staphylococcus aureus* (MRSA)^12,13^. Thus, it is crucial to identify patients with risk factors for these pathogens when deciding the empirical treatment for CAP patients^8^.

The prevalence of CAP due to *Pseudomonas aeruginosa* has increased during the last decade worldwide, especially in LMICs, where prevalence is between 3–5%^14^. Moreover, patients with severe CAP, chronic pulmonary diseases (including tracheostomy), and those with prior infections due to *Pseudomonas aeruginosa* might need empirical antipseudomonal treatment^15^. The American Thoracic Society and the Infectious Diseases Society of America (ATS/IDSA) clinical guidelines recommend using cefepime, piperacillin/tazobactam, or antipseudomonal carbapenems in these patients. Due to stewardship programs and to reserve usage of carbapenems for MDRP, the most frequently used treatment for ICU-admitted CAP patients at risk of *Pseudomonas aeruginosa* infection are cefepime and piperacillin/tazobactam. However, it is unknown which antimicrobial is the best alternative for CAP patients at higher risk for *Pseudomonas aeruginosa* CAP admitted to the ICU^9,11,16^. Moreover, it remains unclear which factors may be associated with choosing cefepime or piperacillin/tazobactam in clinical practice. This study will attempt to bring novel information about mortality outcomes by simulating a randomized controlled trial comparing these two antimicrobial regimens in CAP patients admitted to the ICU.

## Methods

### Data source

This is a retrospective analysis of patients admitted to the ICU and registered in the Medical Information Mart for Intensive Care IV (MIMIC-IV) database. This retrospective cohort study was carried out following the strengthening of the reporting of observational studies in epidemiology (STROBE) guidelines^17^ and the tenets of the Helsinki declaration. The data was taken from the multi-parametric intelligent monitoring data from the ICU at the Beth Israel Deaconess Medical Centre (BIDMC) in Boston, Massachusetts, containing the complete information of 69,639 patients admitted to the ICU between 2008 and 2019^18^. The Massachusetts Institute of Technology and Beth Israel Deaconess Medical Center approved the establishment of the MIMIC-IV database, and written informed consent was obtained to collect raw data. The Laboratory for Computational Physiology (LCP) of the Massachusetts Institute of Technology (MIT) created the database. The MIMIC-IV is supported by the National Institute of Biomedical Imaging and Bioengineering (NIBIB) of the National Institutes of Health (NIH)^18,19^. Further information about the database can be found elsewhere (https://lcp.mit.edu/mimic). All personal information was anonymized, and only a random code was used to identify specific patients. Therefore, the requirement for informed consent and ethical approval was waived by the Ethics Committee of Clinica Universidad de La Sabana (20,230,803) and Universidad de La Sabana (519).

The following variables were obtained during the first 24 h of admission: demographic data, comorbidities (i.e., Charlson comorbidity index), physiological data (i.e., urine output and vital signs), laboratory results (i.e., arterial blood gases, blood count, electrolytes, and renal function), and the requirement of invasive interventions. Clinical outcomes were determined at hospital discharge. All the data was taken directly from the critical care information systems, the electronic hospital records file, laboratory results, and the vital signs monitors, as has been extensively described elsewhere^18,19^. All information was secured with read-only access to ensure data integrity. The MIMIC-IV database is integrated with the US Social Security System to allow access to mortality data even after hospital discharge^18,19^.

### Participants

The cohort only included patients admitted to the ICU due to CAP. The definition of CAP was based on the diagnostic criteria proposed by the ATS/IDSA guidelines^8^. The inclusion criteria were patients older than 18 years, requiring admission to the ICU with an ICD-9/ICD-10 code of pneumonia within the top ten diagnoses in the discharge note, as published previously^20^. Moreover, patients must have received cefepime or piperacillin/tazobactam, which started during the first 24 h of ICU admission and must be continued for at least 72 h. Patients with infectious diagnoses other than pneumonia and those with ventilator-associated pneumonia were excluded. Finally, patients transferred from other institutions, those with less than 70% of the numerical data (i.e., labs and vital signs), and those receiving cefepime and piperacillin/tazobactam at the same time were excluded from this analysis.

### Study groups

The cohort was divided into two groups, based on the antibiotic initiated during the first 24 h of admission: cefepime or piperacillin/tazobactam-based treatments. The usage of other antibiotics recommended by the IDSA/ATS guidelines was registered if the patients fulfilled the inclusion and exclusion criteria specified above.

### Statistical analysis

Continuous variables were described as minimum or maximum values, mean and standard deviation [SD], or median and interquartile range [IQR], depending on their clinical relevance and normality distribution. Dichotomous variables were presented as frequencies and percentages. For the univariate analysis, differences between the intervention groups were assessed with the chi-square test and Fisher’s exact test for categorical variables or the Student's t-test or Mann–Whitney U test for continuous variables, depending on their distribution.

A multivariate logistic regression model was developed to evaluate the factors associated with using cefepime or piperacillin/tazobactam (i.e., dependent variable) and demographics, comorbid conditions, and laboratory variables (i.e., explanatory variables). The logistic regression model included variables with a *p* < 0.20 in the initial bivariate analysis^21^. Odds ratios (OR) were calculated based on the exponentials of the coefficients obtained by the final model and presented in forest plots. A Cox proportional hazard model was constructed for ICU, hospital, and 28-day mortality adjusted by confounders. The adjustment of the survival analysis included the development of septic shock, ventilatory support initiated during the first 24 h of ICU admission, disease severity (i.e., SAPS II), morbidity score (i.e., Charlson), and age variables. Adjusted hazard ratios (HR) were calculated and presented in forest plot charts. An upset plot was constructed to show the other antibiotics used among cefepime or piperacillin/tazobactam. Finally, we performed a targeted maximum likelihood estimation (TMLE) analysis to simulate a randomized controlled trial to estimate the causal effect between exposure to cefepime or piperacillin/tazobactam and ICU, in-hospital and 28-day mortality. We applied a weighting technique using the inverse of the propensity score. This approach was employed to achieve balance in the baseline characteristics of our study groups. By doing so, we aimed to avoid incorporating variables solely linked to the intervention or as intermediate variables along the causal pathway between the initial exposure and the eventual outcome. A significance level of 0.05 and a confidence level of 95% were chosen. Data analysis was done using R version 4.3.1 and SPSS (IBM) version 29.

### Ethical approval

The establishment of the MIMIC-IV database was approved by the Massachusetts Institute of Technology and Beth Israel Deaconess Medical Center and written informed consent was obtained for the collection of raw data. Medical Information Mart for Intensive Care IV (MIMIC-IV) is a freely available medical data for research that has compiled anonymized data from patients treated in Beth Israel Deaconess Medical Centre (BIDMC) in Boston, the privacy rights of human subjects were always observed. Therefore, the requirement for informed consent and ethical approval for this study was waived by the Ethics Committee of Clinica Universidad de La Sabana (20,230,803) and Universidad de La Sabana (519). All procedures for this analysis were carried out following the strengthening of the reporting of observational studies in epidemiology (STROBE) guidelines^19^ and the tenets of the Helsinki declaration.

## Results

**Figure 1:**
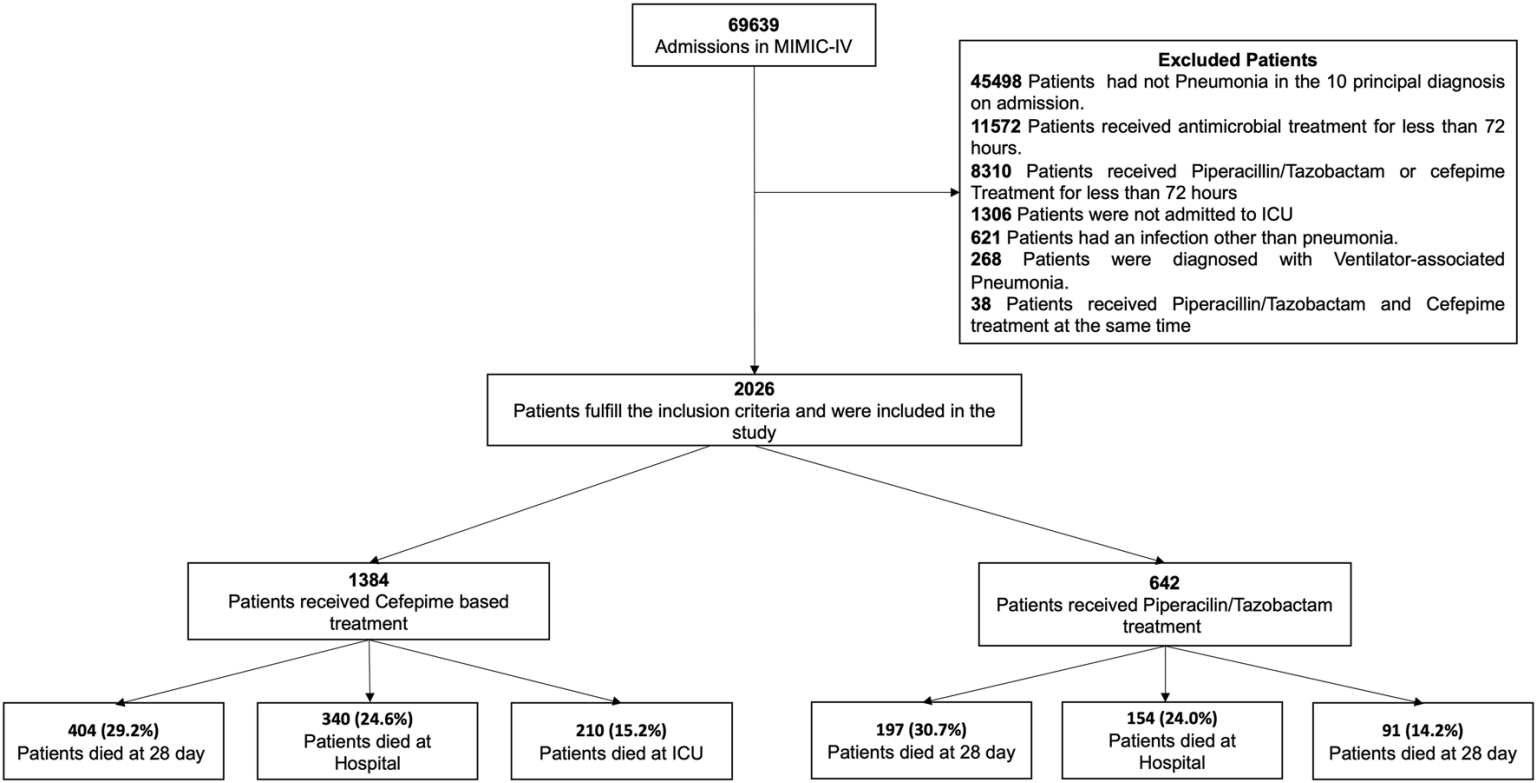
Study flow chart.

A total of 2026 patients were included in the primary analysis (Fig. 1). Male patients represent the major proportion of the cohort, 58.9% (1193/2026), and the mean (SD) age was 68.0 (15.44) years old. The most frequent comorbidities were congestive heart failure 43% (873/2026) and chronic pulmonary disease 41.1% (839/2026), followed by renal disease 27.9% (565/2026), cancer 24.4% (495/2026), and diabetes 23.6% (479/2026). All comorbidities and demographic data are listed in Table 1. Almost 46.7% (947/2026) of patients developed respiratory failure during the first 24 h of ICU admission, and a quarter developed septic shock 26.8% (543/2026). All interventions are shown in Table 1.

**Table 1:** Demographic characteristics of all patients and stratified between treatments.

Characteristic	All Cohort (n = 2026)	Cefepime (n = 1384)	Piperacillin/tazobactam (n = 642)	*p-value*
Demographic				
Male, n (%)	1193 (58.9)	796 (57.5)	397 (61.8)	0.07
Age, mean (SD)	68.0 (15.4)	68.8 (15.0)	66.1 (16.1)	< 0.001
Charlson comorbidity index, mean (SD)	6.8 (2.9)	6.9 (2.9)	6.5 (2.9)	0.003
Laboratory variables at admission, mean (SD)				
Haematocrit, %	29.7 (6.5)	29.8 (6.6)	29.4 (6.4)	0.27
Hemoglobin, mg/dL	9.6 (2.2)	9.6 (2.2)	9.6 (2.2)	0.59
Platelets, cell/mm^3^	209.0 (131.1)	202.5 (126.1)	223.2 (140.5)	0.005
WBC, cell/mm^3^	12.0 (11.5)	11.8 (10.8)	12.5 (12.9)	0.038
Lymphocytes, cell/mm^3^	1.5 (6.9)	1.7 (8.3)	1.1 (1.5)	0.14
Neutrophils, cell/mm^3^	10.7 (6.6)	10.5 (6.7)	11.2 (6.4)	0.026
Anion gap, mEq/L	13.5 (3.6)	13.5 (3.6)	13.5 (3.6)	0.67
Bicarbonate, mEq/L	22.1 (5.7)	22.2 (5.6)	22.0 (6.0)	0.72
BUN, mg/dL	29.6 (22.8)	30.2 (23.2)	28.4 (22.0)	0.1
Calcium, mEq/L	7.9 (0.9)	8.0 (0.9)	7.9 (1.0)	0.033
Chloride, mEq/L	100.3 (7.0)	100.3 (6.9)	100.3 (7.1)	0.88
Creatinine, md/dL	1.5 (1.4)	1.5 (1.4)	1.4 (1.5)	0.69
Glucose, mg/dL	119.2 (45.1)	120.7 (45.0)	116.0 (45.2)	0.009
Sodium, mEq/L	136.5 (5.7)	136.4 (5.7)	136.5 (5.9)	0.9
Potassium, mEq/L	3.9 (0.6)	3.9 (0.6)	3.8 (0.6)	0.003
INR	1.6 (0.8)	1.6 (0.8)	1.5 (0.7)	0.1
PT, sec	16.9 (8.5)	17.1 (9.0)	16.6 (7.4)	0.4
PTT, sec	33.4 (11.7)	33.7 (12.5)	32.9 (9.9)	0.55
Physiological variables at admission, mean (SD)				
Heart rate, BPM	75.8 (16.2)	75.8 (16.0)	75.9 (16.5)	0.85
Systolic blood pressure, mmHg	88.1 (16.1)	88.8 (16.2)	86.6 (15.7)	0.002
Diastolic blood pressure, mmHg	45.1 (11.0)	45.1 (11.0)	45.0 (11.0)	0.76
Median blood pressure, mmHg	56.4 (13.1)	56.7 (13.0)	55.7 (13.4)	0.2
Respiratory rate	14.0 (4.1)	14.2 (4.0)	13.8 (4.3)	0.048
Temperature, °C	36.4 (0.7)	36.4 (0.7)	36.3 (0.8)	0.003
SPO_2_, %	89.9 (6.7)	89.8 (6.4)	90.0 (7.3)	0.16
Urine output, ml	1675.3 (1134.5)	1700.8 (1152.8)	1620.4 (1092.9)	0.24
Urine uutput > 1680, ml n (%)	844 (41.7)	585 (42.3)	259 (40.3)	0.44
Comorbidities, n (%)				
Myocardial infarction	384 (19.0)	271 (19.6)	113 (17.6)	0.32
Congestive heart failure	873 (43.1)	633 (45.7)	240 (37.4)	< 0.001
Peripheral vascular disease	196 (9.7)	138 (10.0)	58 (9.0)	0.56
Cerebrovascular Disease	235 (11.6)	151 (10.9)	84 (13.1)	0.18
Dementia	101 (5.0)	66 (4.8)	35 (5.5)	0.58
Chronic pulmonary disease	839 (41.4)	584 (42.2)	255 (39.7)	0.32
Rheumatic disease	67 (3.3)	51 (3.7)	16 (2.5)	0.21
Peptic ulcer disease	57 (2.8)	34 (2.5)	23 (3.6)	0.2
Mild liver disease	325 (16.0)	197 (14.2)	128 (19.9)	0.001
Severe liver disease	126 (6.2)	73 (5.3)	53 (8.3)	0.013
Diabetes	479 (23.6)	341 (24.6)	138 (21.5)	0.14
Complicated diabetes	190 (9.4)	135 (9.8)	55 (8.6)	0.44
Paraplegia	79 (3.9)	56 (4.0)	23 (3.6)	0.71
Renal disease	565 (27.9)	406 (29.3)	159 (24.8)	0.037
Malignant cancer	495 (24.4)	347 (25.1)	148 (23.1)	0.35
Metastatic solid tumour	238 (11.7)	161 (11.6)	77 (12.0)	0.87
AIDS	30 (1.5)	23 (1.7)	7 (1.1)	0.43
Severity Index at ICU admission, mean (SD)				
SAPS II	41.6 (13.3)	41.3 (12.5)	42.2 (14.8)	0.51
Interventions, n (%)				
HFNC	51 (2.5)	37 (2.7)	14 (2.2)	0.61
Invasive ventilation	813 (40.1)	509 (36.8)	304 (47.4)	< 0.001
Non-invasive ventilation	63 (3.1)	47 (3.4)	16 (2.5)	0.34
Tracheostomy	25 (1.2)	15 (1.1)	10 (1.6)	0.49
Complications, n (%)				
Respiratory failure	947 (46.7)	633 (45.7)	314 (48.9)	0.2
Septic shock	543 (26.8)	352 (25.4)	191 (29.8)	0.047
ARDS	19 (0.9)	15 (1.1)	4 (0.6)	0.45
Outcomes, n (%)				
ICU mortality	301 (14.9)	210 (15.2)	91 (14.2)	0.6
Hospital mortality	494 (24.4)	340 (24.6)	154 (24.0)	0.82
28 days mortality	601 (29.7)	404 (29.2)	197 (30.7)	0.53
90 days mortality	825 (40.7)	548 (39.6)	277 (43.1)	0.14

*BPM* beats per minute; *mmHg* millimeters of mercury; *BUN* Blood urea nitrogen; *WBC* white blood cells; *INR* International normalized ratio; *PT* Prothrombin Time; *PTT* Partial thromboplastin Time; *AIDS* Acquired immunodeficiency syndrome; *SAPS II* Simplified acute physiology score II; *HFNC* High flow nasal cannula; *ARDS* Acute respiratory distress syndrome.

**Figure 2:**
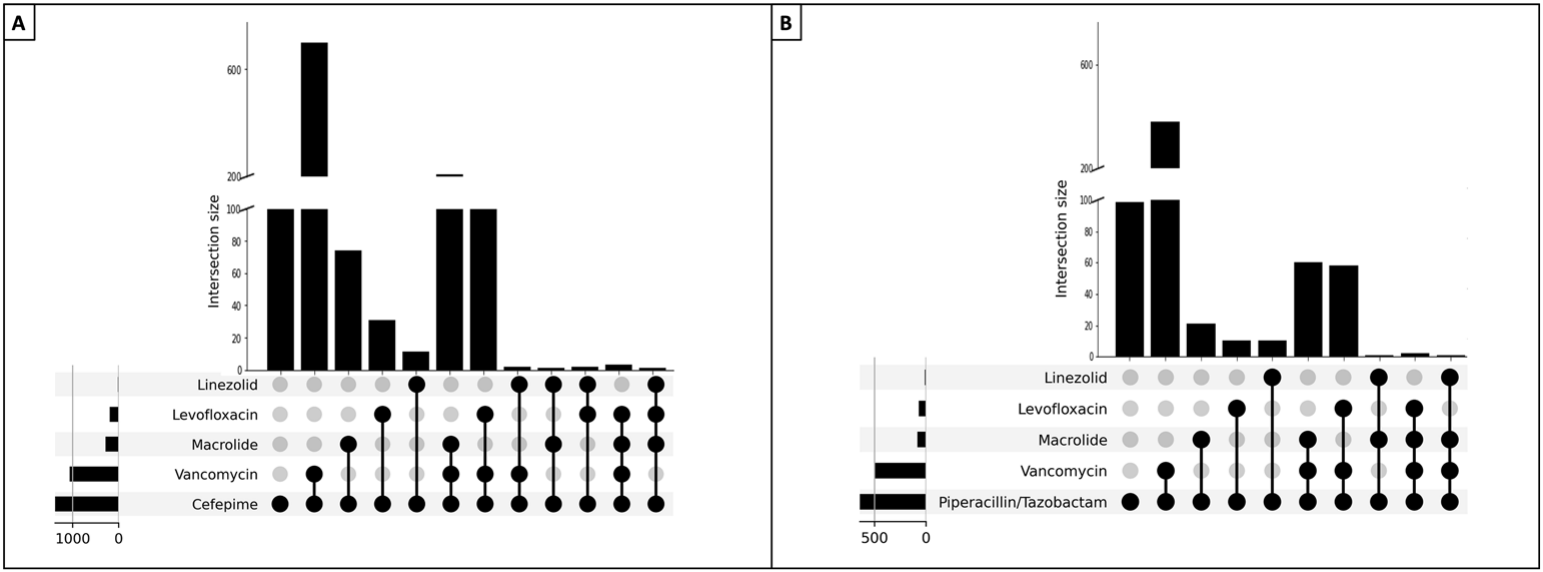
Upset plots antimicrobial combinations.

Only 24% (482/2026) of the cohort had an identified microbiological pathogen. The most frequently identified microorganisms were *Staphylococcus aureus* (36% [173/482]), *Pseudomonas aeruginosa* (18% [88/482]), and *Klebsiella pneumoniae* (9.3% [45/482]) (Fig. E1; Table E1). We identified that the most common concomitant prescription was cefepime or piperacillin/tazobactam plus Vancomycin, followed by triple therapy with cefepime or piperacillin/tazobactam plus vancomycin and macrolide or levofloxacin, which is in concordance with international guidelines. Notably, over half of the patients in each group received vancomycin as the second antimicrobial (Fig. 2).

The cohort was divided into two groups based on the antibiotic treatment (i.e., cefepime or piperacillin/tazobactam) started for CAP during the first 24 h of hospital admission; 68.3% (1384/2026) received cefepime, and 31.7% (642/2026) received piperacillin/tazobactam-based treatment. Patients in the cefepime group were older (mean [SD], 68.8 [15.0] vs. 66.1 [16.1], *p* < 0.001) and had higher Charlson Comorbidity Index score (mean [SD], 6.9 points [2.9] vs 6.5 [2.9], *p* = 0.003). In contrast, patients treated with piperacillin/tazobactam were more frequently males (57.5% [796/1384] vs 61.8% [397/642], *p* = 0.07). Regarding the laboratory results, we found that the white blood count (11.8 [10.8] vs. 12.5 [12.9] *p* = 0.038), neutrophils (10.5 [6.7] vs. 11.2 [6.4] *p* = 0.026), and platelets (202.5 [126.1] vs. 223.2 [140.5] *p* = 0.005), were higher in the piperacillin/tazobactam group. Nonetheless, these values were near physiological ranges and had no clinical relevance (Table 1).

### Comorbid conditions and disease severity on ICU admission

Congestive heart failure and chronic renal disease were more frequently reported in cefepime-treated patients (45.7% [633/1384] vs. 37.4% [240/642] *p* < *0.001*), and (29.3% [406/1384] vs. 24.8% [159/642] *p* = *0.037*), respectively. In contrast, mild liver disease and severe liver disease were higher in the piperacillin/tazobactam group (14.2% [197/1384] vs. 19.9% [128/642] *p* = *0.001*) and (5.3% [73/1384] vs. 8.3% [53/642] *p* = *0.013*). The disease severity was assessed with the SAPS II at ICU admission. Notably, we did not find differences in the SAPS II between the two groups (mean [SD]; 41.3 [12.5] vs 42.2 [14.8] *p* = *0.51*). However, a larger number of patients required invasive mechanical ventilation in the piperacillin/tazobactam group (36.8% [509/1384] vs. 47.4% [304/642], *p* < *0.01*). Other advanced ventilatory supports, such as High-flow nasal cannula (HFNC) or non-invasive ventilation, were not different among the study groups (Table 1).

### Multivariate analysis for factors associated with the use of cefepime or piperacillin/tazobactam

We found that the Age (OR 1.14 95% CI [1.01–1.27], *p* = 0.03), serum potassium (OR 1.14 95% CI [1.03–1.26]), *p* = 0.009), PTT (OR 1.1 95% CI [1.01–1.22], *p* = 0.039) and temperature (OR 1.13 95% CI [1.03–1.24], *p* = 0.014), collected during the first 24 h of hospital admission were associated with preferring cefepime over piperacillin/tazobactam. In contrast, we found that the platelet count (OR 0.80 95% CI [0.72–0.89], *p* < 0.001) and male gender (OR 0.90 95% CI [0.82–9,99], *p* = 0.027) were associated with choosing piperacillin/tazobactam over cefepime. Interestingly, the SAPS II, respiratory failure, septic shock, or ARDS were not associated with choosing the antibiotic (Table E2; Fig. E2).

### Clinical outcomes

Almost one-third of the patients died at 28 days (29.7% [601/2026]). Moreover, the 90-day mortality was 40.7% (825/2026) (Table 1). Notably, a larger number of patients in the group of piperacillin/tazobactam present septic shock during the ICU admission (29.8% [191/642] *p* = *0.047* vs. 25.4% [352/1384]) (Table 1). When analyzing mortality among the study groups, we found that the 28-day mortality (29.2% [404/1384] vs 30.7% [197/642], *p* = *0.53*), hospital mortality (24.6% [340/1384] vs. 24% [154/642], *p* = *0.82*) and the ICU mortality (15.2% [210/ 1384] vs. 14.2% [91/642], *p* = *0.6*), were similar among the study groups (Table 1; Fig. E3).

**Figure 3:**
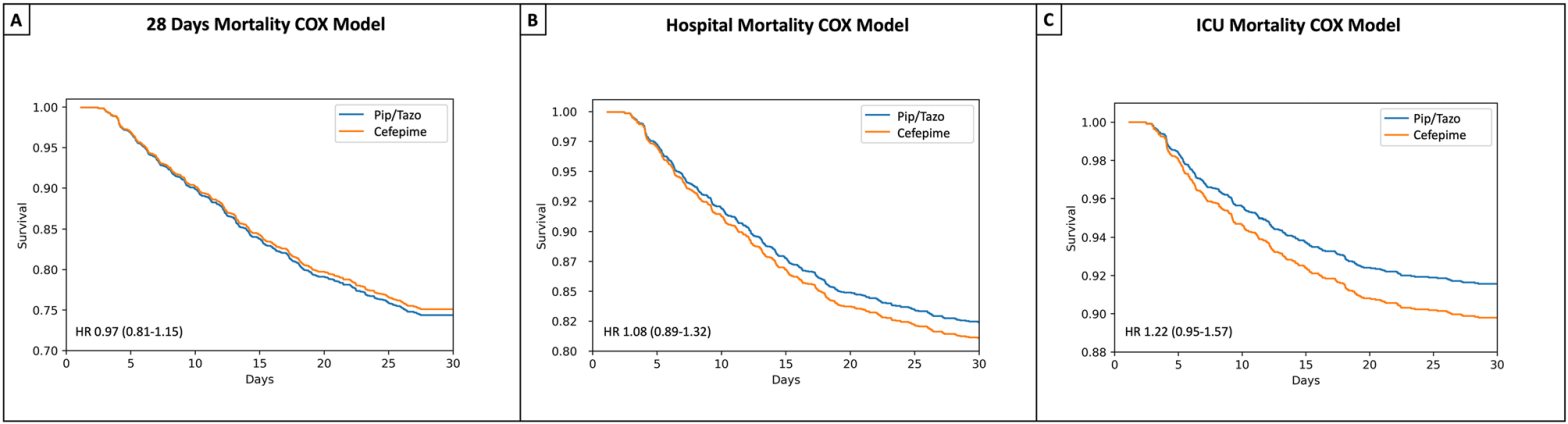
Survival models. Cox Proportional Hazard Curves to identify factors associated with **A** 28-day mortality, **B** Hospital mortality, and **C** ICU Mortality.

### Survival analysis

The COX proportional hazard model analysis (Fig. 3) did not find differences in the adjusted risk for 28 days, hospital and ICU mortality when the patients were treated with cefepime (28 days: HR [95% CI] 0.97 [0.81–1.15]; hospital: 1.08 [0.89–1.32]; ICU: 1.22 [0.95–1.57]) compared to treated with piperacillin/tazobactam. The Cox proportional hazard regression output is shown in an additional file (Fig. E4).

**Figure 4:**
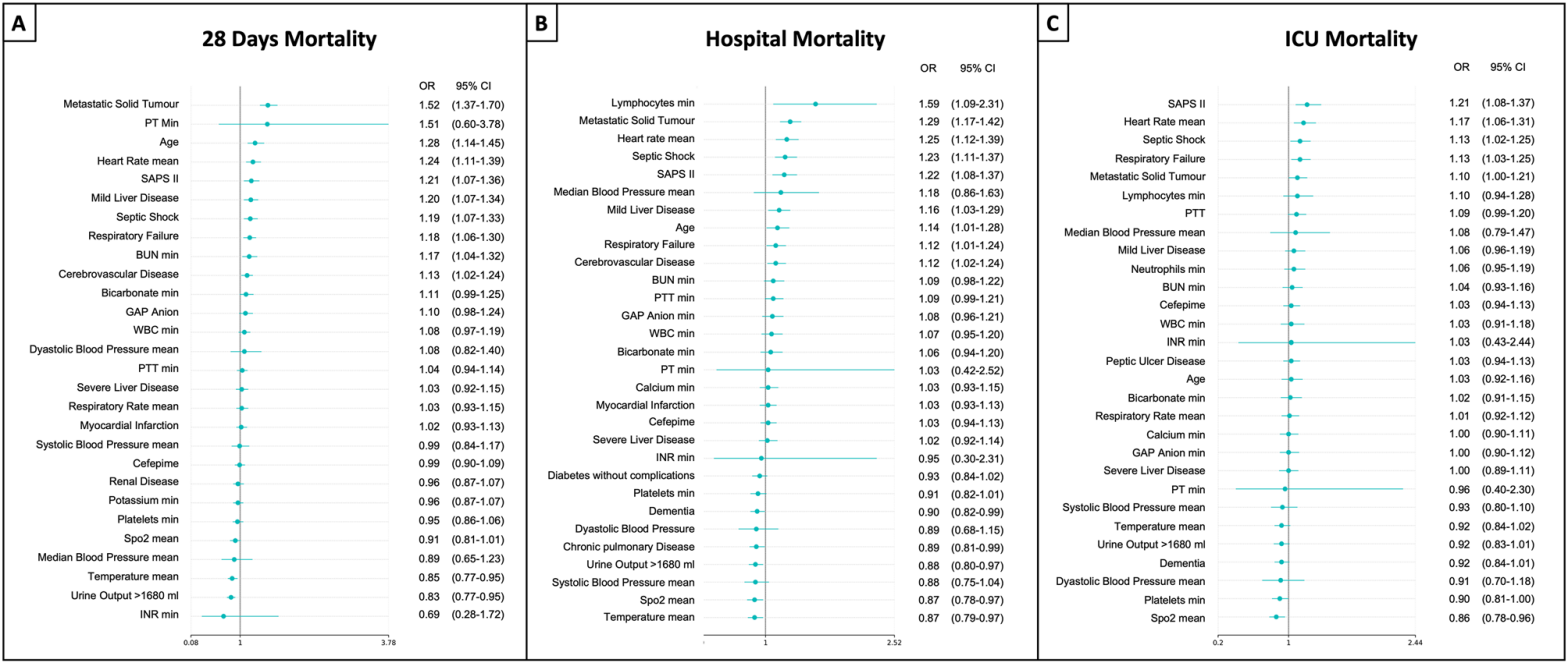
Multivariate logistic regression model for mortality. **A** 28-day mortality **B** Hospital mortality **C** ICU Mortality.

### Multivariate analysis for mortality between patients cefepime versus piperacillin/tazobactam treatment

**Figure 5:**
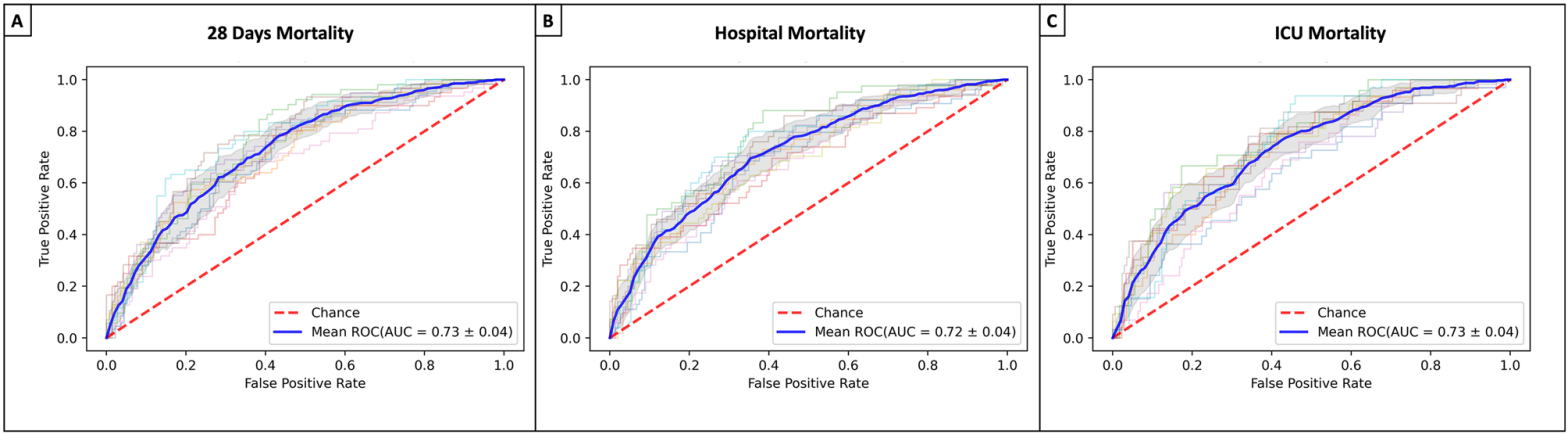
The area under the Curve. Cross-validation trial's receiver operative curve (ROC) for the subset of the selected variables. The blue curve represents the average of the ROC curves of each test, and the average area under the ROC is also presented**. A** shows the AUC-ROC for 28 days mortality **B** for Hospital mortality** C** ICU Mortality.

After adjusting for confounding variables, a logistic regression model was performed for 28 days, hospital and ICU mortality (Fig. 4; Tables E3, E4, E5). Neither cefepime nor piperacillin/tazobactam was identified as a risk factor nor protective for 28 days, hospital and ICU mortality. The model used had a good discriminatory capacity when evaluated by the AUROC, with a mean (SD) of 0.74 (0.03) for 28 days, hospital and ICU mortality (Fig. 5).

### Targeted maximum likelihood estimation (TMLE) analysis.

Finally, a TMLE analysis was performed, and no association between cefepime or piperacillin/tazobactam with 28 days, hospital and ICU mortality was found. The p-values were greater than 0.05, and the additive effect *p*-value of 95% CI were 28 days mortality (0.50193 [ − 0.039 to 0.019]), hospital mortality (0.338 [ − 0.012 to 0.037]), ICU mortality (0.181 [ − 0.006 to 0.037]), confirming that there are no differences in the clinical outcomes among the study groups.

## Discussion

This study compared cefepime and piperacillin/tazobactam-based antimicrobial treatments in patients admitted to the ICU due to CAP. We found no significant differences in 28 days, hospital, and ICU mortality among patients treated with cefepime or piperacillin/tazobactam, even though we found a greater prevalence of septic shock development and IMV requirement during ICU stay in patients treated with piperacillin/tazobactam. Additionally, congestive heart and renal diseases were found more frequently in the cefepime group, while liver diseases were more prevalent in the piperacillin/tazobactam group. The most common concomitant prescription was cefepime or piperacillin/tazobactam plus vancomycin, followed by triple therapy with cefepime or piperacillin/tazobactam plus vancomycin and macrolide or levofloxacin. To our knowledge, these findings are novel because while there is a wide range of information about empirical antimicrobials discussed in the ATS/IDSA guidelines, there is little information about which recommended antimicrobials are best for critically ill CAP patients with a higher risk for *Pseudomonas aeruginosa* infection.

The optimal empirical antimicrobial regimen for CAP patients admitted to the ICU remains unclear. Previous studies have compared the use of cefepime and piperacillin/tazobactam in various infectious diseases^22–25^. Recently, Qian ET et al*.* published the results of the ACORN study, finding that mortality was comparable among patients treated with cefepime and piperacillin/tazobactam; however, this study included a heterogeneous group of patients with different clinical diagnoses, not only CAP. Thus, there is no information comparing the clinical outcomes of patients treated with cefepime or piperacillin/tazobactam for CAP in patients admitted to ICU. Our study of CAP patients admitted to the ICU did not find substantial differences in mortality, this complements the limited available data.

The choice of empirical antimicrobials in patients diagnosed with CAP depends on several factors, including age, local etiological epidemiology, comorbidities, suspected causative microorganisms, risk factors for multidrug-resistant pathogens, and the treating physician’s criteria^26–28^. The factors associated with cefepime or piperacillin/tazobactam choice are not widely explored, and there is not enough data on which specific population it is preferred to use cefepime or piperacillin/tazobactam in clinical practice. In our study, we did find that cefepime usage was preferred in aged patients with renal impairment comorbidities and congestive heart failure. At the same time, piperacillin/tazobactam was more frequently used in patients with liver comorbidities and low systolic blood pressure values. The latter can explain why there was a major prevalence of septic shock development and IMV requirement in the piperacillin/tazobactam group. Concerning renal function and the use of beta-lactams such as cefepime or piperacillin/tazobactam, it is important to mention that their association with vancomycin increases the risk of acute kidney injury as described by Bellos et al.in their systematic review of 2020^29^. Although adverse events related to the development of acute kidney injury such as neurotoxicity are more frequent with cefepime, the risk of renal injury is higher in patients treated with piperacillin/tazobactam and vancomycin^29,30^. Our findings are interesting as prior data have shown that patients with abnormal renal function treated with cefepime are at a higher risk of neurotoxicity^30^. Notably, the FDA has a warrant suggesting the dosage adjustment in patients with creatinine clearance less than or equal to 60 mL/min in these patients^31^. Clinicians should assess if there is past medical evidence of renal injury when choosing of beta-lactam empirically therapy and make appropriate dose adjustments to avoid complications.

Finally, we find that common concomitant antimicrobial prescription was cefepime or piperacillin/tazobactam plus vancomycin, followed by triple therapy with cefepime or piperacillin/tazobactam plus vancomycin and macrolide or levofloxacin. This finding reflects the adoption of IDSA/ATS guidelines and recommendations for critically ill patients^8^. Even when Gram-positive sensitive pathogens usually cause CAP, guidelines suggest a broad-spectrum antibiotic empiric management in patients who require ICU^8^. In addition, dual therapy usually ensures coverage against atypical germs, improves the likelihood of initial adequate treatment, shortens the length of stay, and improves survival^20,32–34^. It is essential to point out that the prevalence of *Pseudomonas aeruginosa* as a causing pathogen of CAP in this cohort is 4.3% (88/2026) in all included patients and 18.2% (88/482) in those who had some identified micro-organisms which are comparable to the data generated in a multinational point prevalence study by Restrepo et al.where they found a prevalence of 4.2% (133/3193) in the whole cohort and 11.3% (133/1173) among the culture-positive patients^14^. This reinforces the importance of antipseudomonal antibiotics in high-risk patients, as are those with severe CAP. On the other hand, the wide usage of vancomycin is striking, causing some authors to propose using narrow-spectrum antimicrobials^35^. Additionally, the MRSA prevalence in CAP is less than 4%^36,37^.

Our study has limitations and strengths that must be recognized. First, this is a monocentric, observational, non-randomized study design. However, we included an extensive sample of patients admitted to the ICU over ten years, improving the results' generalizability. Moreover, we performed a robust statistical approach using TMLE to simulate a randomized clinical trial, adjusted survival analyses, and multivariate regression models to compare cefepime vs. piperacillin/tazobactam. To better understand the cohorts, we also performed multivariate models to identify the factors associated with prescribing cefepime or piperacillin-tazobactam in ICU-admitted CAP patients. Second, patient data were collected in a high-income country, making it difficult to extrapolate and replicate the methodology to validate this data in low- and middle-income countries. However, cefepime and piperacillin/tazobactam are widely used and inexpensive antimicrobial regimens globally. Third, no standardized protocols of antimicrobial treatment, doses, start time, and total days of administration were used, which also restricted the stratification analysis by these data. Moreover, sensitivity analysis of the groups without vancomycin or linezolid was not performed. Nevertheless, international guidelines with dosing recommendations are known globally, frequently used in ICU patients, and suggest combined antimicrobial treatments. Finally, the susceptibility data of the pathogens is not available. However, this study focuses on the comparison of empirical and non-targeted treatments; also, cefepime and piperacillin/tazobactam are two broad-spectrum antimicrobials whose effectiveness against gram-negative germs, especially *Pseudomonas aeruginosa*, are equivalent.

In conclusion, our study used robust statistical analysis to compare two empirical antimicrobial regimens for patients with CAP at higher risk of *Pseudomonas aeruginosa* and the factors associated with their prescription in patients admitted to the ICU. Although this study did not demonstrate differences in mortality between the two groups, it corroborates that using cefepime and piperacillin/tazobactam in ICU-admitted CAP patients could be equivalent, and clinicians could establish their preferences availability and security profile. Additional prospective studies are required to support these conclusions.

### Supplementary Information

Supplementary Information.

## Data Availability

Available by request to the corresponding authors.
