# Supplementary material for: A targeted likelihood estimation comparing cefepime and piperacillin/tazobactam in critically ill patients with community-acquired pneumonia (CAP)
Source: Sci Rep. 2024 Jun 11;14:13392. doi: 10.1038/s41598-024-64444-3 (PMC11166966; doi:10.1038/s41598-024-64444-3)
Supplement: Supplementary file 1 — Supplementary Information. [file 41598_2024_64444_MOESM1_ESM.docx]

**TITLE:** A targeted likelihood estimation comparing cefepime and piperacillin/tazobactam in critically ill patients with Community-Acquired Pneumonia (CAP).

**AUTHORS:** Cristian C. Serrano-Mayorga MD^1,2,3,4#^, Sara Duque MSc^1#^, Elsa D. Ibáñez-Prada MD^1,2,3^, Esteban Garcia-Gallo PhD ^1,10^, María P Rojas Arrieta MD ^2^, Alirio Bastidas MSc^2^, Alejandro Rodríguez PhD ^5^, Ignacio Martin-Loeches PhD ^6,7,8,9##^ and Luis F. Reyes PhD^1,2,3,10*##^.

# Co-first authors. (CCSM and SD contributed equally to this manuscript)

## Co-senior authors.

**AFFILIATIONS:** 1, Unisabana Center for Translational Science, Universidad de La Sabana, Chía, Colombia; 2, School of Medicine, Universidad de La Sabana, Chía, Colombia; 3, Clinica Universidad de La Sabana, Chía, Colombia; 4, Bioscience Ph.D., Engineering faculty, Universidad de La Sabana, Chía, Colombia; 5, ICU Hospital Universitario de Tarragona Joan XXIII – IISPV – Universidad Rovira & Virgili - CIBERES, Tarragona, Spain; 6, Department of Intensive Care Medicine, Multidisciplinary Intensive Care Research Organisation (MICRO), St James's Hospital, Dublin, Ireland; 7, Trinity College Dublin, Dublin, Ireland; 8, CIBER of Respiratory Diseases (CIBERES), Institute of Health Carlos III, Madrid, Spain; 9, Pulmonary Department, Hospital Clinic, Universitat de Barcelona, IDIBAPS, ICREA, Barcelona, Spain; 10, Pandemic Sciences Institute, University of Oxford, Oxford, United Kingdom.

**CORRESPONDING AUTHOR:** Luis Felipe Reyes, MD., PhD., Universidad de La Sabana, Chía, Colombia. Phone number: 57 861 55 55 Ext: 23342. Email: [luis.reyes5@unisabana.edu.co](mailto:luis.reyes5@unisabana.edu.co)

**Table of Contents**

**Tables**

**Table E1.** Microorganisms stratified by groups.

**Table E2.** Univariate analysis and logistic regression for using Cefepime or Piperacillin/Tazobactam.

**Table E3.** Univariate and multivariate analysis for mortality 28 days.

**Table E4.** Univariate and multivariate analysis for Hospital mortality.

**Table E5.** Univariate and multivariate analysis for ICU mortality.

**Figures**

**Figure E1.** Pneumonia causal agents. **Panel A** Shows the most frequent causative microorganisms of pneumonia, and **Panel C** "other" specified.

**Figure E2.** Multivariate logistic regression model for using Cefepime or Piperacillin/Tazobactam.

**Figure E3.** Comparative figure between mortality among Cefepime and Piperacillin/Tazobactam groups **Panel A.** 28 days Mortality **Panel B.** Hospital Mortality **Panel C.** ICU Mortality.

**Figure E4.** Cox Proportional Hazard Regression was used to identify factors associated with 28-day, hospital and ICU mortality.

**TABLES**

**Table E1.** Microorganisms stratified by groups.

| **Microorganism** | **Whole Cohort (n=482)** | **Cefepime (n=283)** | **Piperacillin/ Tazobactam (n=199)** |  |
| --- | --- | --- | --- | --- |
|  |  |  |  |  |
| *A. baumannii* | 14 (2.9%) | 8 (2.83%) | 6 (3.02%) |  |
| *A. hydrophila* | 1 (0.21%) | 0 (0%) | 1 (0.5%) |  |
| *Acinetobacter spp.* | 2 (0.41%) | 1 (0.35%) | 1 (0.5%) |  |
| *Alcaligenes spp.* | 2 (0.41%) | 1 (0.35%) | 1 (0.5%) |  |
| *Aspergillus spp.* | 22 (4.56%) | 11 (3.89%) | 11 (5.53%) |  |
| *B. cepacia* | 4 (0.83%) | 3 (1.06%) | 1 (0.5%) |  |
| *C. koseri* | 2 (0.41%) | 1 (0.35%) | 1 (0.5%) |  |
| *E. aerogenes* | 7 (1.45%) | 5 (1.77%) | 2 (1.01%) |  |
| *E. asburiae* | 1 (0.21%) | 0 (0%) | 1 (0.5%) |  |
| *E. cloacae* | 12 (2.49%) | 4 (1.41%) | 8 (4.02%) |  |
| *E. coli* | 28 (5.81%) | 13 (4.59%) | 15 (7.54%) |  |
| *H. alvei* | 1 (0.21%) | 0 (0%) | 1 (0.5%) |  |
| *Haemophilus spp.* | 1 (0.21%) | 0 (0%) | 1 (0.5%) |  |
| *K. oxytoca* | 6 (1.24%) | 3 (1.06%) | 3 (1.51%) |  |
| *K. pneumoniae* | 45 (9.34%) | 21 (7.42%) | 24 (12.06%) |  |
| *M. catarrhalis* | 6 (1.24%) | 4 (1.41%) | 2 (1.01%) |  |
| *M. morganii* | 2 (0.41%) | 0 (0%) | 2 (1.01%) |  |
| *P. aeruginosa* | 88 (18.26%) | 59 (20.85%) | 29 (14.57%) |  |
| *P. jirovecii* | 1 (0.21%) | 0 (0%) | 1 (0.5%) |  |
| *P. stuartii* | 2 (0.41%) | 0 (0%) | 2 (1.01%) |  |
| *S. aureus* | 173 (35.89%) | 108 (38.16%) | 65 (32.66%) |  |
| *S. maltophila* | 28 (5.81%) | 18 (6.36%) | 10 (5.03%) |  |
| *S. marcescens* | 12 (2.49%) | 6 (2.12%) | 6 (3.02%) |  |
| *S. pneumonia* | 18 (3.73%) | 13 (4.59%) | 5 (2.51%) |  |
| *Corynebacterium spp.* | 1 (0.21%) | 1 (0.35%) | 0 (0%) |  |
| *M. tuberculosis* | 2 (0.41%) | 2 (0.71%) | 0 (0%) |  |
| *Serratia spp.* | 1 (0.21%) | 1 (0.35%) | 0 (0%) |  |

**Table E2.** Univariate analysis and logistic regression for using Cefepime or Piperacillin/Tazobactam.

| **Variable** | **Univariate Analysis** | | **Multivariate Analysis** | |
| --- | --- | --- | --- | --- |
|  | **OR (95% CI)** | ***p-value*** | **OR (95% CI)** | ***p-value*** |
| **Demographic** | | | | |
| Male | 0.83 (0.69-1.01) | 0.07 | 0.90 (0.82-0.99) | **0.027** |
| Age | 1.01 (1.00-1.02) | **<0.001** | 1.14 (1.01-1.27) | **0.03** |
| Charlson Comorbidity Index | 1.05 (1.01-1.08) | **0.004** | 1.07 (0.95-1.21) | 0.28 |
| **Laboratory variables at admission** | | | | |
| Hematocrit, % | 1.01 (0.99-1.02) | 0.2 |  |  |
| Hemoglobin ; mg/dl | 1.01 (0.97-1.06) | 0.56 |  |  |
| Platelets, cell/mm^3^ | 1.00 (1.00-1.00) | **0.001** | 0.80 (0.72-0.89) | **<0.001** |
| WBC, cell/mm^3^ | 0.99 (0.99-1.00) | 0.23 |  |  |
| Lymphocytes, cell/mm^3^ | 1.05 (0.99-1.10) | 0.1 | 1.08 (0.95-1.22) | 0.25 |
| Neutrophils, cell/mm^3^ | 0.99 (0.97-1.00) | **0.042** | 0.97 (0.88-1.07) | 0.5 |
| Anion GAP, mEq/L | 0.99 (0.97-1.02) | 0.71 |  |  |
| Bicarbonate, mEq/L | 1.00 (0.99-1.02) | 0.6 |  |  |
| Bun, mg/dL | 1.00 (1.00-1.01) | 0.11 | 1.03 (0.92-1.15) | 0.57 |
| Calcium, mEq/L | 1.10 (1.00-1.23) | **0.06** | 0.99 (0.89-1.09) | 0.8 |
| Chloride, mEq/L | 1.00 (0.98-1.01) | 0.87 |  |  |
| Creatinine, md/dL | 1.00 (0.94-1.08) | 0.88 |  |  |
| Glucose, mg/dL | 1.00 (1.00-1.00) | **0.03** | 1.03 (0.94-1.14) | 0.49 |
| Sodium, mEq/L | 1.00 (0.98-1.01) | 0.81 |  |  |
| Potassium, mEq/L | 1.26 (1.08-1.48) | **0.003** | 1.14 (1.03-1.26) | **0.009** |
| INR | 1.08 (0.95-1.22) | 0.23 |  |  |
| PT | 1.01 (1.00-1.02) | 0.2 |  |  |
| PTT | 1.01 (1.00-1.01) | 0.18 | 1.11 (1.01-1.22) | **0.039** |
| **Physiological Variables at Admission** | | | | |
| Heart Rate mean, BPM | 1.00 (0.99-1.00) | 0.24 |  |  |
| Systolic Blood Pressure mean, mmHg | 1.01 (1.00-1.01) | **0.005** | 1.08 (0.92-1.26) | 0.34 |
| Diastolic Blood Pressure, mmHg | 1.00 (0.99-1.01) | 0.82 |  |  |
| Median Blood Pressure mean, mmHg | 1.01 (1.00-1.01) | 0.12 | 1.02 (0.90-1.14) | 0.8 |
| Respiratory Rate | 1.03 (1.00-1.05) | **0.03** | 1.06 (0.94-1.19) | 0.36 |
| Temperature, C° | 1.20 (1.06-1.36) | **0.004** | 1.13 (1.03-1.24) | **0.014** |
| SPO2 mean, % | 1.00 (0.98-1.01) | 0.68 |  |  |
| Urine Output, ml | 1.00 (1.00-1.00) | 0.14 | 1.05 (0.95-1.15) | 0.36 |
| **Comorbidities** | | | | |
| Myocardial Infarction | 1.14 (0.89-1.45) | 0.29 |  |  |
| Congestive Heart Failure | 1.41 (1.17-1.71) | **<0.001** | 1.10 (1.00-1.22) | **0.06** |
| Peripheral Vascular Disease | 1.11 (0.81-1.54) | 0.51 |  |  |
| Cerebrovascular Disease | 0.81 (0.61-1.08) | 0.16 | 0.93 (0.85-1.02) | 0.12 |
| Dementia | 0.87 (0.57-1.32) | 0.51 |  |  |
| Chronic Pulmonary Disease | 1.11 (0.92-1.34) | 0.29 |  |  |
| Rheumatic Disease | 1.50 (0.85-2.65) | 0.16 | 1.03 (0.94-1.13) | 0.5 |
| Peptic Ulcer Disease | 0.68 (0.40-1.16) | 0.16 | 0.95 (0.87-1.04) | 0.29 |
| Mild Liver Disease | 0.67 (0.52-0.85) | **0.001** | 0.90 (0.80-1.00) | **0.048** |
| Severe Liver Disease | 0.62 (0.43-0.89) | **0.01** | 0.94 (0.84-1.04) | 0.23 |
| Diabetes | 1.19 (0.95-1.50) | 0.12 | 1.03 (0.93-1.13) | 0.58 |
| Complicated Diabetes | 1.15 (0.83-1.60) | 0.39 |  |  |
| Paraplegia | 1.14 (0.69-1.86) | 0.62 |  |  |
| Renal Disease | 1.26 (1.02-1.56) | **0.033** | 1.00 (0.89-1.12) | 0.96 |
| Cancer | 1.12 (0.90-1.39) | 0.32 |  |  |
| AIDS | 1.53 (0.65-3.59) | 0.32 |  |  |
| Metastatic Solid Tumor | 0.97 (0.72-1.29) | 0.81 |  |  |
| **Severity Index at UCI admission** | | | | |
| SAPS II | 0.99 (0.99-1.00) | 0.14 | 0.91 (0.80-1.02) | 0.11 |
| **Complications** | | | | |
| Respiratory Failure | 0.88 (0.73-1.06) | 0.18 | 0.96 (0.87-1.05) | 0.36 |
| Septic Shock | 0.81 (0.65-0.99) | 0.041 | 1.00 (0.90-1.10) | 0.92 |
| ARDS | 1.75 (0.58-5.29) | 0.32 |  |  |
| BPM: Beats per minute, mmHG: milimiters of mercury BUN: Blood Urea Nitrogen; WBC: white blood cells; INR: International Normalized Ratio; PT: Prothrombin Time; PTT: Partial Thromboplastin Time; AIDS: Acquired Immunodeficiency Syndrome; SAPS II: Simplified Acute Physiology Score II; HFNC: High Flow Nasal Cannula; ARDS: Acute respiratory distress syndrome. | | | | |

**Table E3.** Univariate and multivariate analysis for mortality 28 days.

| **Variable** | **Univariate Analysis** | | **Multivariate Analysis** | |
| --- | --- | --- | --- | --- |
|  | **p-value** | **OR (95% CI)** | **p-value** | **OR (95% CI)** |
| **Demographic** | | | | |
| Male | 0.23 | 1.13 (0.93-1.37) |  |  |
| Age | <0.001 | 1.02 (1.01-1.03) | **<0.001** | 1.28 (1.14-1.45) |
| Cefepime | 0.49 | 0.93 (0.76-1.14) | 0.83 | 0.99 (0.90-1.09) |
| **Laboratory variables at admission** | | | | |
| Platelets, cell/mm^3^ | 0.06 | 1.00 (1.00-1.00) | 0.35 | 0.95 (0.86-1.06) |
| WBC, cell/mm^3^ | 0.034 | 1.01 (1.00-1.02) | 0.16 | 1.08 (0.97-1.19) |
| Lymphocytes, cell/mm^3^ | 0.38 | 1.01 (0.99-1.02) |  |  |
| Neutrophils, cell/mm^3^ | 0.21 | 1.01 (0.99-1.02) |  |  |
| Anion GAP, mEq/L | <0.001 | 1.07 (1.04-1.10) | 0.1 | 1.10 (0.98-1.24) |
| Bicarbonate, mEq/L | 0.005 | 0.98 (0.96-0.99) | 0.08 | 1.11 (0.99-1.25) |
| Bun, mg/dL | <0.001 | 1.01 (1.01-1.02) | 0.011 | 1.17 (1.04-1.32) |
| Calcium, mEq/L | 0.62 | 1.03 (0.92-1.14) |  |  |
| Chloride, mEq/L | 0.91 | 1.00 (0.99-1.01) |  |  |
| Creatinine, mg/dL | 0.28 | 1.04 (0.97-1.11) |  |  |
| Glucose, mg/dL | 0.55 | 1.00 (1.00-1.00) |  |  |
| Sodium, mEq/L | 0.35 | 1.01 (0.99-1.02) |  |  |
| Potassium, mEq/L | 0.2 | 1.11 (0.95-1.29) | 0.42 | 0.96 (0.87-1.06) |
| INR | 0.003 | 1.19 (1.06-1.33) | 0.43 | 0.69 (0.28-1.72) |
| PT | 0.002 | 1.02 (1.01-1.03) | 0.38 | 1.51 (0.60-3.78) |
| PTT | 0.018 | 1.01 (1.00-1.02) | 0.48 | 1.04 (0.94-1.14) |
| **Physiological Variables at Admission** | | | | |
| Heart Rate, BPM | <0.001 | 1.01 (1.01-1.02) | **<0.001** | 1.24 (1.11-1.39) |
| Systolic Blood Pressure, mmHg | <0.001 | 0.99 (0.98-0.99) | 0.89 | 0.99 (0.84-1.17) |
| Diastolic Blood Pressure, mmHg | 0.003 | 0.99 (0.98-0.99) | 0.6 | 1.08 (0.82-1.40) |
| Median Blood Pressure, mmHg | <0.001 | 0.98 (0.97-0.99) | 0.5 | 0.89 (0.65-1.23) |
| Respiratory Rate | 0.19 | 1.01 (0.99-1.04) | 0.53 | 1.03 (0.93-1.15) |
| Temperature, C° | <0.001 | 0.67 (0.57-0.79) | **0.002** | 0.85 (0.77-0.95) |
| SPO2, % | 0.036 | 0.96 (0.93-1.00) | **0.07** | 0.91 (0.81-1.01) |
| Urine Output, ml | <0.001 | 0.51 (0.42-0.63) | **<0.001** | 0.83 (0.75-0.91) |
| **Comorbidities** | | | | |
| Myocardial Infarction | 0.17 | 1.18 (0.93-1.50) | 0.63 | 1.02 (0.93-1.13) |
| Congestive Heart Failure | 0.84 | 1.02 (0.84-1.24) |  |  |
| Cerebrovascular Disease | 0.06 | 1.31 (0.99-1.75) | **0.016** | 1.13 (1.02-1.24) |
| Dementia | 0.26 | 1.27 (0.83-1.94) |  |  |
| Chronic Pulmonary Disease | 0.44 | 0.93 (0.76-1.12) |  |  |
| Rheumatic Disease | 0.61 | 0.87 (0.50-1.50) |  |  |
| Peptic Ulcer Disease | 0.54 | 1.19 (0.68-2.08) |  |  |
| Mild Liver Disease | 0.001 | 1.53 (1.20-1.96) | **0.002** | 1.20 (1.07-1.34) |
| Severe Liver Disease | 0.02 | 1.55 (1.07-2.25) | 0.66 | 1.03 (0.92-1.15) |
| Diabetes | 0.72 | 0.96 (0.77-1.20) |  |  |
| Complicated Diabetes | 0.92 | 1.02 (0.73-1.41) |  |  |
| Paraplegia | 0.39 | 0.80 (0.48-1.33) |  |  |
| Renal Disease | <0.001 | 3.17 (2.41-4.18) | **<0.001** | 1.52 (1.37-1.70) |
| AIDS | 0.4 | 1.38 (0.65-2.92) |  |  |
| Metastatic Solid Tumor | <0.001 | 2.36 (1.78-3.13) | **<0.001** | 1.29 (1.17-1.42) |
| **Severity Index at admission** | | | | |
| SAPS II | <0.001 | 1.04 (1.03-1.05) | **0.003** | 1.21 (1.07-1.36) |
| **Outcomes** | | | | |
| Respiratory Failure | <0.001 | 1.62 (1.34-1.97) | **0.002** | 1.18 (1.06-1.30) |
| Septic Shock | <0.001 | 2.02 (1.64-2.48) | **0.001** | 1.19 (1.07-1.33) |
| ARDS | 0.85 | 1.09 (0.41-2.90) |  |  |
| BPM: Beats per minute, mmHG: milimiters of mercury BUN: Blood Urea Nitrogen; WBC: white blood cells; INR: International Normalized Ratio; PT: Prothrombin Time; PTT: Partial Thromboplastin Time; AIDS: Acquired Immunodeficiency Syndrome; SAPS II: Simplified Acute Physiology Score II; HFNC: High Flow Nasal Cannula; ARDS: Acute respiratory distress syndrome. | | | | |

**Table E4.** Univariate and multivariate analysis for Hospital mortality.

| **Variable** | **Univariate Analysis** | | **Multivariate Analysis** | |
| --- | --- | --- | --- | --- |
|  | **p-value** | **OR (95% CI)** | **p-value** | **OR (95% CI)** |
| **Demographic** | | | | |
| Male | 0.25 | 0.89 (0.72-1.09) |  |  |
| Age | 0.002 | 1.01 (1.00-1.02) | **0.036** | 1.14 (1.01-1.28) |
| Cefepime | 0.78 | 1.03 (0.83-1.28) | 0.56 | 1.03 (0.94-1.13) |
| **Laboratory variables at admission** | | | | |
| Platelets, cell/mm^3^ | <0.001 | 1.00 (1.00-1.00) | 0.07 | 0.91 (0.82-1.01) |
| WBC, cell/mm^3^ | 0.005 | 1.01 (1.00-1.02) | 0.3 | 1.07 (0.95-1.20) |
| Lymphocytes, cell/mm^3^ | 0.015 | 1.02 (1.00-1.04) | **0.016** | 1.59 (1.09-2.31) |
| Neutrophils, cell/mm^3^ | 0.41 | 1.01 (0.99-1.02) |  |  |
| Anion GAP, mEq/L | <0.001 | 1.07 (1.04-1.10) | 0.2 | 1.08 (0.96-1.21) |
| Bicarbonate, mEq/L | <0.001 | 0.96 (0.94-0.98) | 0.31 | 1.06 (0.94-1.20) |
| Bun, mg/dL | <0.001 | 1.01 (1.01-1.01) | 0.11 | 1.09 (0.98-1.22) |
| Calcium, mEq/L | 0.13 | 0.92 (0.82-1.03) | 0.56 | 1.03 (0.93-1.15) |
| Chloride, mEq/L | 0.5 | 1.00 (0.99-1.02) |  |  |
| Creatinine, mg/dL | 0.56 | 1.02 (0.95-1.10) |  |  |
| Glucose, mg/dL | 0.58 | 1.00 (1.00-1.00) |  |  |
| Sodium, mEq/L | 0.48 | 1.01 (0.99-1.02) |  |  |
| Potassium, mEq/L | 0.81 | 0.98 (0.83-1.16) |  |  |
| INR | 0.025 | 1.14 (1.02-1.28) | 0.92 | 0.95 (0.39-2.31) |
| PT | 0.02 | 1.01 (1.00-1.02) | 0.94 | 1.03 (0.42-2.52) |
| PTT | 0.001 | 1.01 (1.01-1.02) | 0.08 | 1.09 (0.99-1.21) |
| **Physiological Variables at Admission** | | | | |
| Heart Rate, BPM | <0.001 | 1.02 (1.01-1.02) | **<0.001** | 1.25 (1.12-1.39) |
| Systolic Blood Pressure, mmHg | <0.001 | 0.98 (0.97-0.99) | 0.14 | 0.88 (0.75-1.04) |
| Diastolic Blood Pressure, mmHg | 0.037 | 0.99 (0.98-1.00) | 0.37 | 0.89 (0.68-1.15) |
| Median Blood Pressure, mmHg | 0.006 | 0.98 (0.97-1.00) | 0.3 | 1.18 (0.86-1.63) |
| Respiratory Rate | 0.32 | 1.01 (0.99-1.04) |  |  |
| Temperature, C° | 0.001 | 0.75 (0.63-0.89) | **0.01** | 0.87 (0.79-0.97) |
| SPO2 , % | 0.009 | 0.95 (0.91-0.99) | **0.01** | 0.87 (0.78-0.97) |
| Urine Output, ml | <0.001 | 0.55 (0.45-0.69) | **0.011** | 0.88 (0.80-0.97) |
| **Comorbidities** | | | | |
| Myocardial Infarction | 0.17 | 1.19 (0.93-1.53) | 0.58 | 1.03 (0.93-1.13) |
| Congestive Heart Failure | 0.67 | 1.05 (0.85-1.28) |  |  |
| Cerebrovascular Disease | 0.06 | 1.34 (0.99-1.81) | **0.016** | 1.12 (1.02-1.24) |
| Dementia | 0.12 | 0.66 (0.39-1.11) | **0.032** | 0.90 (0.82-0.99) |
| Chronic Pulmonary Disease | 0.018 | 0.78 (0.63-0.96) | **0.024** | 0.89 (0.81-0.99) |
| Rheumatic Disease | 0.92 | 0.97 (0.55-1.72) |  |  |
| Peptic Ulcer Disease | 0.33 | 1.33 (0.75-2.37) |  |  |
| Mild Liver Disease | <0.001 | 1.75 (1.36-2.27) | **0.01** | 1.16 (1.03-1.29) |
| Severe Liver Disease | 0.001 | 1.86 (1.28-2.72) | 0.68 | 1.02 (0.92-1.14) |
| Diabetes | 0.07 | 0.80 (0.62-1.02) | 0.12 | 0.93 (0.84-1.02) |
| Complicated Diabetes | 0.77 | 1.05 (0.75-1.49) |  |  |
| Paraplegia | 0.74 | 0.91 (0.53-1.56) |  |  |
| Renal Disease | 0.29 | 1.13 (0.90-1.41) |  |  |
| AIDS | 0.89 | 0.94 (0.40-2.21) |  |  |
| Metastatic Solid Tumor | <0.001 | 2.36 (1.78-3.13) | **<0.001** | 1.29 (1.17-1.42) |
| **Severity Index at admission** | | | | |
| SAPS II | <0.001 | 1.04 (1.03-1.05) | **0.002** | 1.22 (1.08-1.37) |
| **Outcomes** | | | | |
| Respiratory Failure | <0.001 | 1.62 (1.32-1.99) | **0.025** | 1.12 (1.01-1.24) |
| Septic Shock | <0.001 | 2.22 (1.79-2.75) | **<0.001** | 1.23 (1.11-1.37) |
| ARDS | 0.84 | 1.11 (0.40-3.09) |  |  |
| BPM: Beats per minute, mmHG: milimiters of mercury BUN: Blood Urea Nitrogen; WBC: white blood cells; INR: International Normalized Ratio; PT: Prothrombin Time; PTT: Partial Thromboplastin Time; AIDS: Acquired Immunodeficiency Syndrome; SAPS II: Simplified Acute Physiology Score II; HFNC: High Flow Nasal Cannula; ARDS: Acute respiratory distress syndrome. | | | | |

**Table E5.** Univariate and multivariate analysis for ICU mortality.

| **Variable** | **Univariate Analysis** | | **Multivariate Analysis** | |
| --- | --- | --- | --- | --- |
|  | **p-value** | **OR (95% CI)** | **p-value** | **OR (95% CI)** |
| **Demographic** | | | | |
| Male | 0.88 | 0.98 (0.76-1.26) |  |  |
| Age | 0.021 | 1.01 (1.00-1.02) | 0.6 | 1.03 (0.92-1.16) |
| Cefepime | 0.56 | 1.08 (0.83-1.41) | 0.56 | 1.03 (0.94-1.13) |
| **Laboratory variables at admission** | | | | |
| Platelets, cell/mm^3^ | <0.001 | 1.00 (1.00-1.00) | 0.049 | 0.90 (0.81-1.00) |
| WBC, cell/mm^3^ | 0.01 | 1.01 (1.00-1.02) | 0.62 | 1.03 (0.91-1.18) |
| Lymphocytes, cell/mm^3^ | 0.031 | 1.01 (1.00-1.03) | 0.24 | 1.10 (0.94-1.28) |
| Neutrophils, cell/mm^3^ | 0.037 | 1.02 (1.00-1.04) | 0.29 | 1.06 (0.95-1.19) |
| Anion GAP, mEq/L | 0.005 | 1.05 (1.01-1.08) | 0.96 | 1.00 (0.90-1.12) |
| Bicarbonate, mEq/L | <0.001 | 0.96 (0.94-0.98) | 0.71 | 1.02 (0.91-1.15) |
| Bun, mg/dL | <0.001 | 1.01 (1.00-1.01) | 0.5 | 1.04 (0.93-1.16) |
| Calcium, mEq/L | 0.003 | 0.82 (0.71-0.94) | 0.94 | 1.00 (0.90-1.11) |
| Chloride, mEq/L | 0.57 | 1.00 (0.99-1.02) |  |  |
| Creatinine, mg/dL | 0.44 | 1.03 (0.95-1.12) |  |  |
| Glucose, mg/dL | 0.27 | 1.00 (1.00-1.00) |  |  |
| Sodium, mEq/L | 0.58 | 1.01 (0.98-1.03) |  |  |
| Potassium, mEq/L | 0.8 | 1.03 (0.84-1.25) |  |  |
| INR | 0.05 | 1.14 (1.00-1.30) | 0.96 | 1.03 (0.43-2.44) |
| PT | 0.049 | 1.01 (1.00-1.02) | 0.93 | 0.96 (0.40-2.30) |
| PTT | 0.001 | 1.01 (1.01-1.02) | 0.1 | 1.09 (0.99-1.20) |
| **Physiological Variables at Admission** | | | | |
| Heart Rate, BPM | <0.001 | 1.02 (1.01-1.02) | **0.003** | 1.17 (1.06-1.31) |
| Systolic Blood Pressure, mmHg | <0.001 | 0.98 (0.97-0.98) | 0.4 | 0.93 (0.80-1.10) |
| Diastolic Blood Pressure, mmHg | 0.002 | 0.98 (0.97-0.99) | 0.47 | 0.91 (0.70-1.18) |
| Median Blood Pressure, mmHg | <0.001 | 0.97 (0.96-0.99) | 0.63 | 1.08 (0.79-1.47) |
| Respiratory Rate | 0.07 | 1.03 (1.00-1.05) | 0.82 | 1.01 (0.92-1.12) |
| Temperature, C° | 0.015 | 0.78 (0.64-0.95) | 0.1 | 0.92 (0.84-1.02) |
| SPO2, % | <0.001 | 0.92 (0.88-0.96) | **0.005** | 0.86 (0.78-0.96) |
| Urine Output, ml | <0.001 | 0.53 (0.41-0.69) | **0.07** | 0.92 (0.83-1.01) |
| **Comorbidities** | | | | |
| Myocardial Infarction | 0.64 | 1.08 (0.79-1.46) |  |  |
| Congestive Heart Failure | 0.77 | 1.04 (0.81-1.33) |  |  |
| Cerebrovascular Disease | 0.55 | 1.12 (0.77-1.62) |  |  |
| Dementia | 0.049 | 0.48 (0.23-1.00) | **0.08** | 0.92 (0.84-1.01) |
| Chronic Pulmonary Disease | 0.67 | 1.05 (0.82-1.35) |  |  |
| Rheumatic Disease | 0.72 | 1.13 (0.58-2.18) |  |  |
| Peptic Ulcer Disease | 0.09 | 1.72 (0.92-3.24) | 0.47 | 1.03 (0.94-1.13) |
| Mild Liver Disease | 0.001 | 1.67 (1.23-2.25) | 0.26 | 1.06 (0.96-1.19) |
| Severe Liver Disease | 0.018 | 1.70 (1.10-2.64) | 0.94 | 1.00 (0.89-1.11) |
| Diabetes | 0.86 | 0.97 (0.73-1.30) |  |  |
| Complicated Diabetes | 0.7 | 1.08 (0.72-1.63) |  |  |
| Paraplegia | 0.81 | 0.92 (0.48-1.77) |  |  |
| Renal Disease | 0.88 | 1.02 (0.78-1.34) |  |  |
| AIDS | 0.78 | 1.15 (0.44-3.02) |  |  |
| Metastatic Solid Tumor | 0.001 | 1.78 (1.27-2.48) | **0.04** | 1.10 (1.00-1.21) |
| **Severity Index at admission** | | | | |
| SAPS II | <0.001 | 1.04 (1.03-1.05) | **0.001** | 1.21 (1.08-1.37) |
| **Outcomes** | | | | |
| Respiratory Failure | <0.001 | 2.02 (1.57-2.59) | **0.014** | 1.13 (1.03-1.25) |
| Septic Shock | <0.001 | 2.46 (1.91-3.17) | **0.02** | 1.13 (1.02-1.25) |
| ARDS | 0.91 | 1.07 (0.31-3.71) |  |  |
| BPM: Beats per minute, mmHG: milimiters of mercury BUN: Blood Urea Nitrogen; WBC: white blood cells; INR: International Normalized Ratio; PT: Prothrombin Time; PTT: Partial Thromboplastin Time; AIDS: Acquired Immunodeficiency Syndrome; SAPS II: Simplified Acute Physiology Score II; HFNC: High Flow Nasal Cannula; ARDS: Acute respiratory distress syndrome. | | | | |

**FIGURES**

**Figure E1.** Pneumonia causal agents. **Panel A** Shows the most frequent causative microorganisms of pneumonia, and **Panel B** "other" specified.


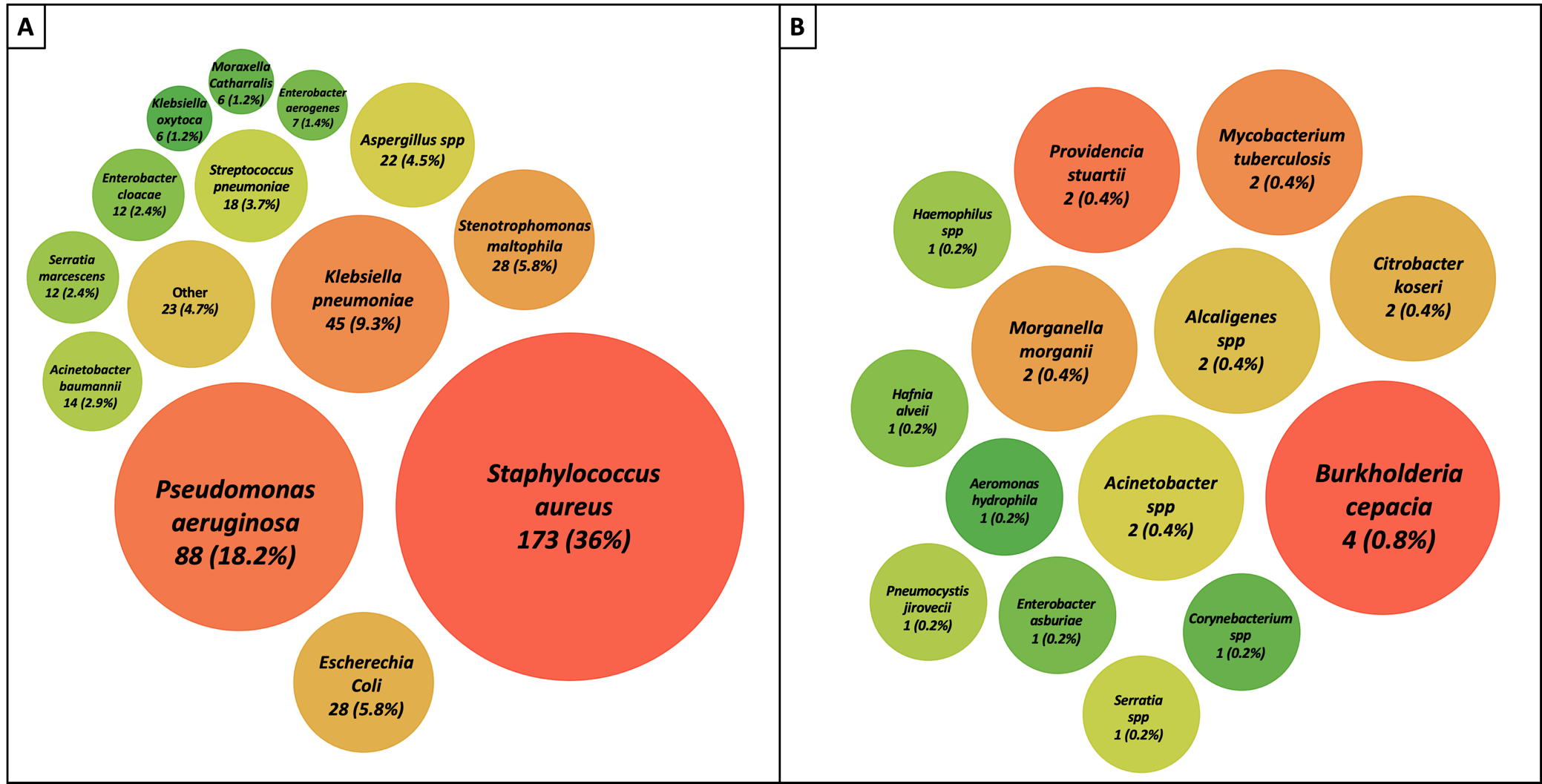


**Figure E2.** Multivariate logistic regression model for using Cefepime or Piperacillin/Tazobactam.

**
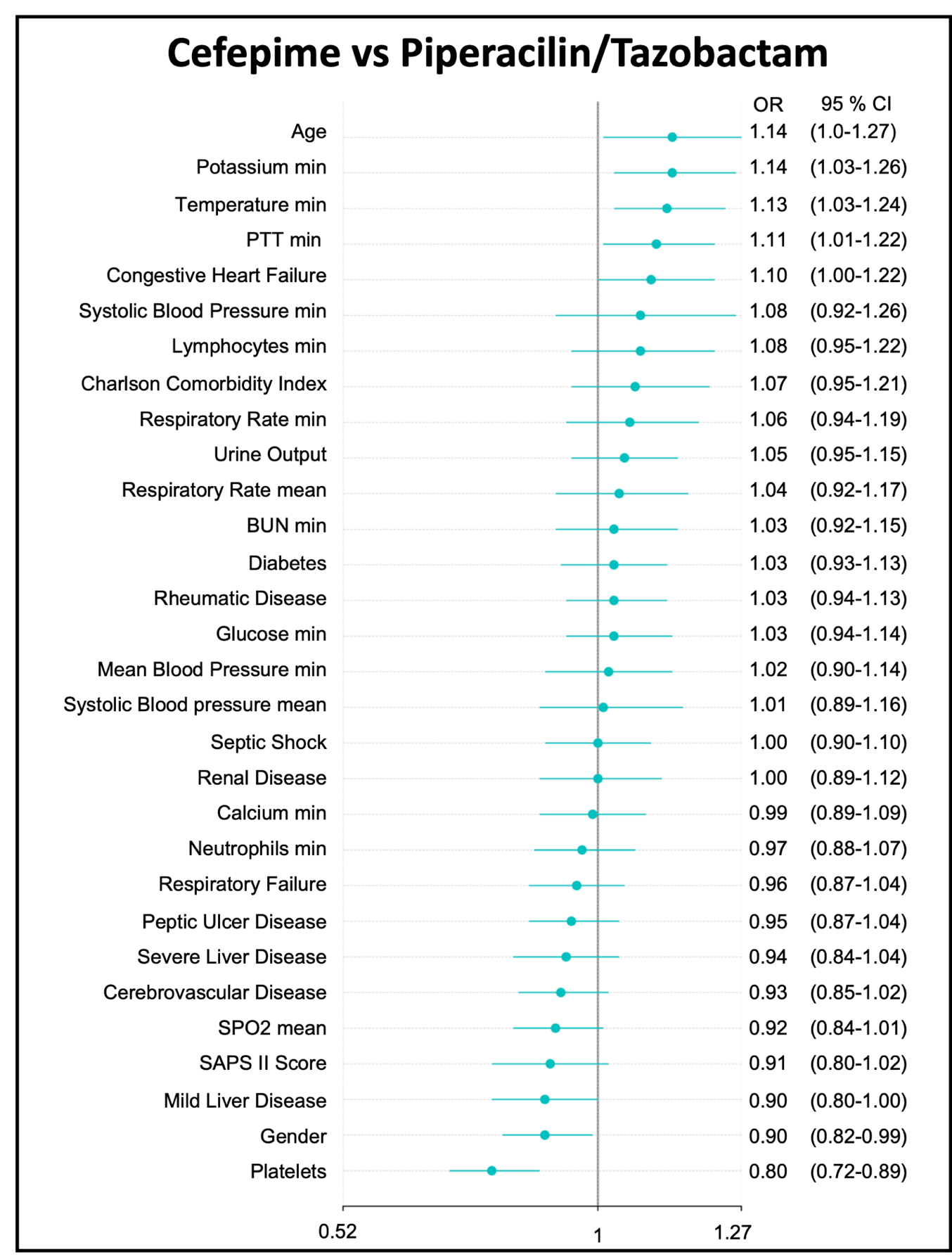
**

**Figure E3.** Comparative figure between mortality among Cefepime and Piperacillin/Tazobactam groups **Panel A.** 28 days Mortality **Panel B.** Hospital Mortality **Panel C.** ICU Mortality.


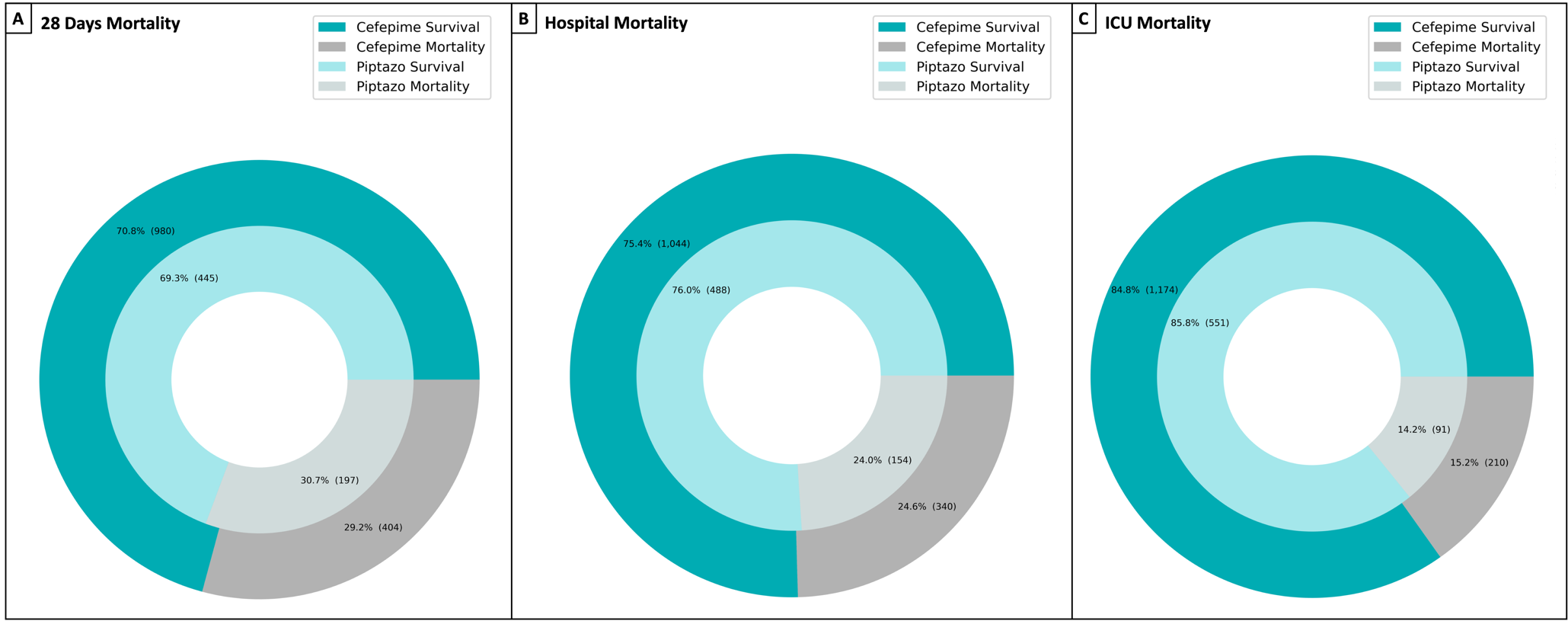


**Figure E4.** Cox Proportional Hazard Regression was used to identify factors associated with 28-day, hospital and ICU mortality.

**
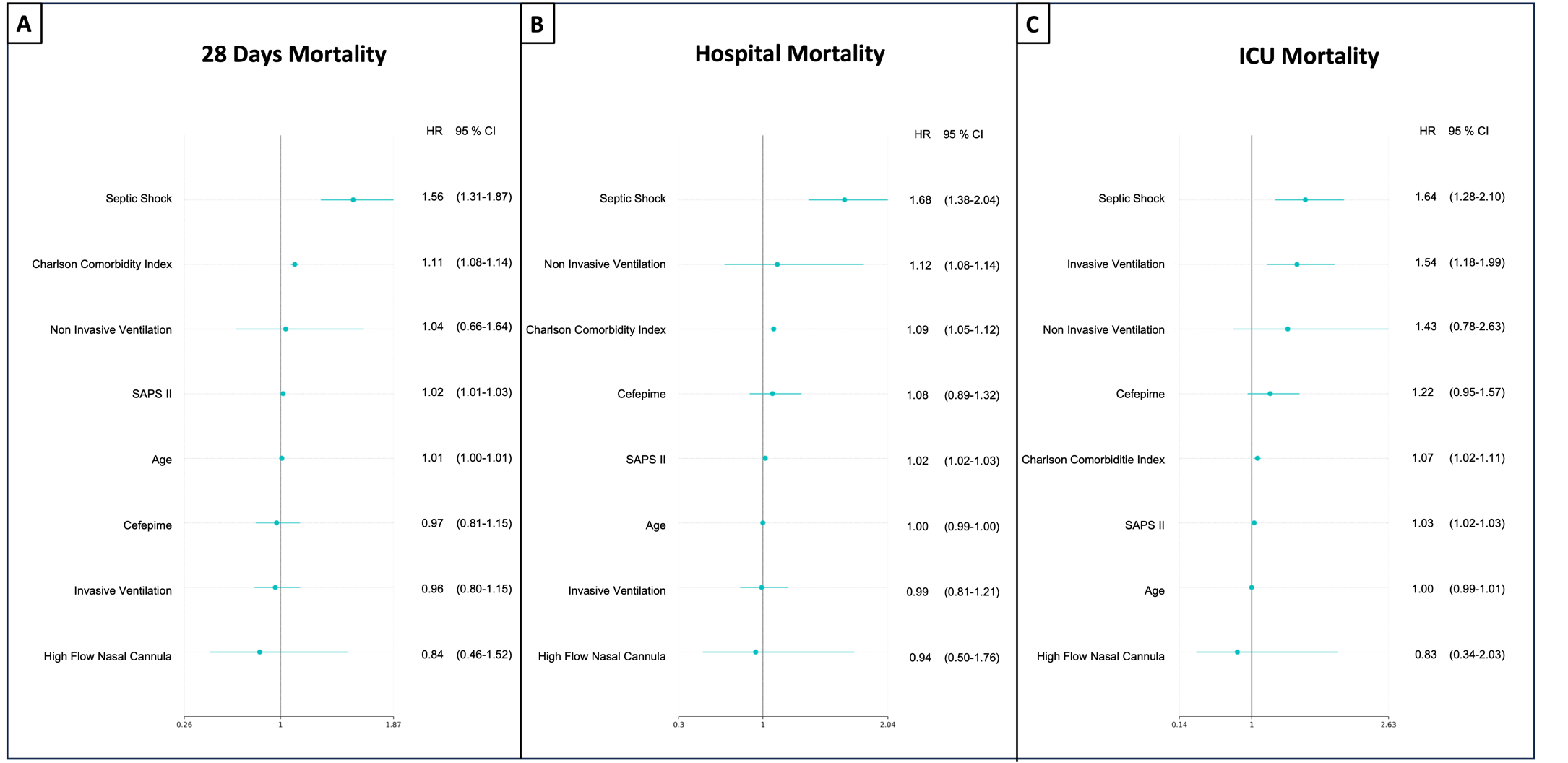
**
